# Prediction and analysis of multiple protein lysine modified sites based on conditional wasserstein generative adversarial networks

**DOI:** 10.1186/s12859-021-04101-y

**Published:** 2021-03-31

**Authors:** Yingxi Yang, Hui Wang, Wen Li, Xiaobo Wang, Shizhao Wei, Yulong Liu, Yan Xu

**Affiliations:** 1 Department of Information and Computer Science, University of Science and Technology Beijing, Beijing, 100083 China; 2 Institute of Computing Technology, Chinese Academy of Sciences, Beijing, 100080 China; 3 No. 15 Research Institute, China Electronics Technology Group Corporation, Beijing, 100083 China

**Keywords:** Post-translational modification, Deep learning, Generative adversarial networks, Random forest

## Abstract

**Background:**

Protein post-translational modification (PTM) is a key issue to investigate the mechanism of protein’s function. With the rapid development of proteomics technology, a large amount of protein sequence data has been generated, which highlights the importance of the in-depth study and analysis of PTMs in proteins.

**Method:**

We proposed a new multi-classification machine learning pipeline MultiLyGAN to identity seven types of lysine modified sites. Using eight different sequential and five structural construction methods, 1497 valid features were remained after the filtering by Pearson correlation coefficient. To solve the data imbalance problem, Conditional Generative Adversarial Network (CGAN) and Conditional Wasserstein Generative Adversarial Network (CWGAN), two influential deep generative methods were leveraged and compared to generate new samples for the types with fewer samples. Finally, random forest algorithm was utilized to predict seven categories.

**Results:**

In the tenfold cross-validation, accuracy (Acc) and Matthews correlation coefficient (MCC) were 0.8589 and 0.8376, respectively. In the independent test, Acc and MCC were 0.8549 and 0.8330, respectively. The results indicated that CWGAN better solved the existing data imbalance and stabilized the training error. Alternatively, an accumulated feature importance analysis reported that CKSAAP, PWM and structural features were the three most important feature-encoding schemes. MultiLyGAN can be found at https://github.com/Lab-Xu/MultiLyGAN.

**Conclusions:**

The CWGAN greatly improved the predictive performance in all experiments. Features derived from CKSAAP, PWM and structure schemes are the most informative and had the greatest contribution to the prediction of PTM.

**Supplementary Information:**

The online version contains supplementary material available at 10.1186/s12859-021-04101-y.

## Background

As a common occurrence in the body, protein-translational modification (PTM) plays an important role in regulating various physiological processes and functions. PTM refers to the process of covalent modification of individual amino acid residues on a protein after the mRNA has been translated into a protein [1]. However, insufficient information restricts the analysis of PTMs to delve deeper. In the past few decades, the advancement of proteomics technology and the development of “Big Data” on protein sequences shed light on the substantial study of protein nature. Although high-throughput biological technology has made tremendous achievements in protein PTM identification and analysis, the conventional approaches require expensive labor but get an unsatisfactory understanding of the relationship between structures and functions. Therefore, it is of paramount significance to develop reliable and efficient computational methods for predicting and analyzing modifications.

Alternatively, protein lysine modifications (PLMs), prevalent PTM types, which occur at active ε-amino groups of specific lysine residues in proteins and are critical for orchestrating various biological processes. So far, a series of computational prediction tools have been developed. These predictors firstly employed feature construction methods including sequences and physicochemical properties. Then, machine learning algorithms were adopted to train models. The published predictors about seven types of lysine modified sites are as follows: (1) Acetylation: NetAcet [2], PAIL [3], BRABSB-PHKA [4], PSKAcePred [5], LAceP [6], N-Ace [7], ASEB [8], ProAcePred [9] and DeepAcet [10]; (2) Glycation: GlyNN [11], PreGly [12], Gly-PseAAC [13], Glypre [14], BPB_GlySite [15], and iProtGly-SS [16]; (3) Succinylation: SucPred [17], iSuc-PseAAC [18], iSuc-PseOpt [19], SuccFind [20], SuccinSite [21], pSuc-Lys [22], SSEvol-Suc [23], and PSuccE [24]; (4) Ubiquitination: UbPred [25], CKSAAP_UbSite [26], UbiProber [27], UbiNet [28] and DeepUbi [29]; (5) SUMO: SUMOpre [30], SUMmOn [31] and seeSUMO [32]; (6) Methylation: AutoMotif Server [33], MASA [34], and PSSMe [35]; (7) Malonylation: MaloPred [36] and Mal-Lys [37]. However, these tools cannot implement classification of all potential lysine modified PTMs, only focusing on a single type, which limits the possibility of mining more information and ignores the interconnections of multiple PTMs.

The data imbalance issue was characterized by prediction bias across widely divergent categories, therefore minimizing the bias is essential for downstream exploration in the prediction of PTMs. Here, we aim to harness deep generative methodology to solve the issue. In 2014, Goodfellow et al. first proposed the Generative Adversarial Nets (GAN) [38]. GAN achieved a great success and directly inspired researchers’ interests in image generation and restoration. Later, it was widely used in various fields, especially image processing and natural language processing [39]. The common generative models based on deep learning ideas include VAE (Variational Auto Encoding), GAN, and variant models of GAN (conditional generative confrontation network (CGAN) [40]: adds the label information as well as Wasserstein Generative Adversarial Network WGAN [41]: completely solved the problem of unstable GAN training). To leverage both advantages, we integrated the CGAN and WGAN to construct the CWGAN for powerful ability of processing data-imbalance in this paper.

**Fig. 1:**
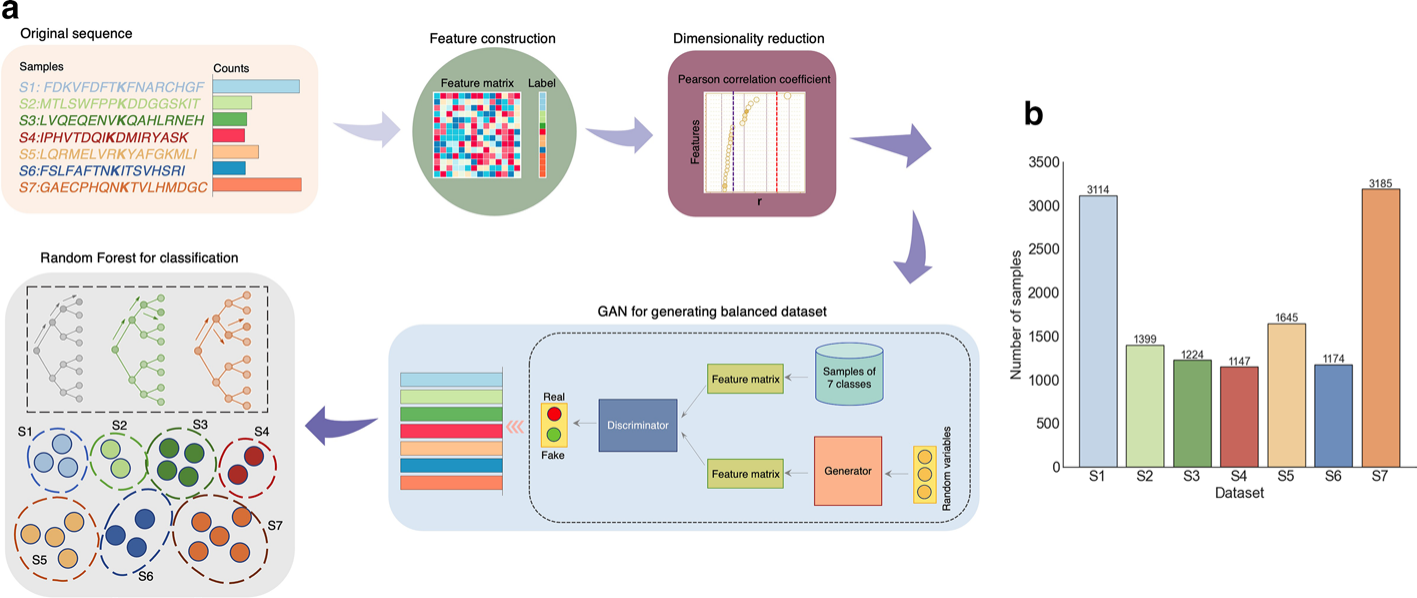
**a** The pipeline of identification of multiple protein modified sites. **b** Sample size distributions within seven types of PTMs, including Acetylation (S1), Glycation (S2), Malonylation (S3), Methylation (S4), Succinylation (S5), Sumoylation (S6) and Ubiquitination (S7)

To further study the underlying mechanisms and the relationship of features and some specific modifications, Random Forest was utilized as a classifier and explain feature importance. The whole pipeline MultiLyGAN is shown in Fig. 1a.

## Results

### Cross-validation results

Among the multiclassification problems, Accuracy (Acc), Confusion Entropy (CEN), Matthews Correlation Coefficient (MCC) and Cross-validation error rate (E_C_) and independent test error rate (E_I_) can be measured to evaluate statistical model performance (Details are shown in Additional file: S4). In this work, 4/5 of all samples were used as training samples (for training the model and cross-validation measurement), and the other 1/5 was utilized as an independent test set. There were 2359 features after eight kinds of sequence-encoding schemes and five structural-encoding schemes. High correlations within the features may weaken the prediction performance, resulting in low prediction accuracy, increase training difficulty and over-fitting risk. Thus, Pearson correlation coefficient (PCC) was calculated between each feature and labels, and further, we discarded features with an absolute value of the PCC greater than 0.5.

In the tenfold cross-validation of training samples, after PCC, the Acc increased by 5%; the MCC increased by 0.057; CEN and E_C_ decreased significantly (Table 1). The remaining features after PCC played a more effective role, implying the deleted features have a negative effect on the prediction results. Moreover, the performance was clearly improved after CGAN where MCC reached 0.8114, and E_C_ was reduced by nearly 2 times after CGAN in Table 1.

**Table 1:** Comparisons of tenfold cross-validation results after PCC, CGAN and CWGAN

	Acc	MCC	CEN	E_C_
*Before PCC*	0.6448	0.5663	0.4229	0.3552
*After PCC*	0.6906	0.6237	0.3859	0.3094
*After CGAN*	0.8365	0.8114	0.2500	0.1635
*After CWGAN*	0.8589	0.8376	0.2219	0.1411

**Fig. 2:**
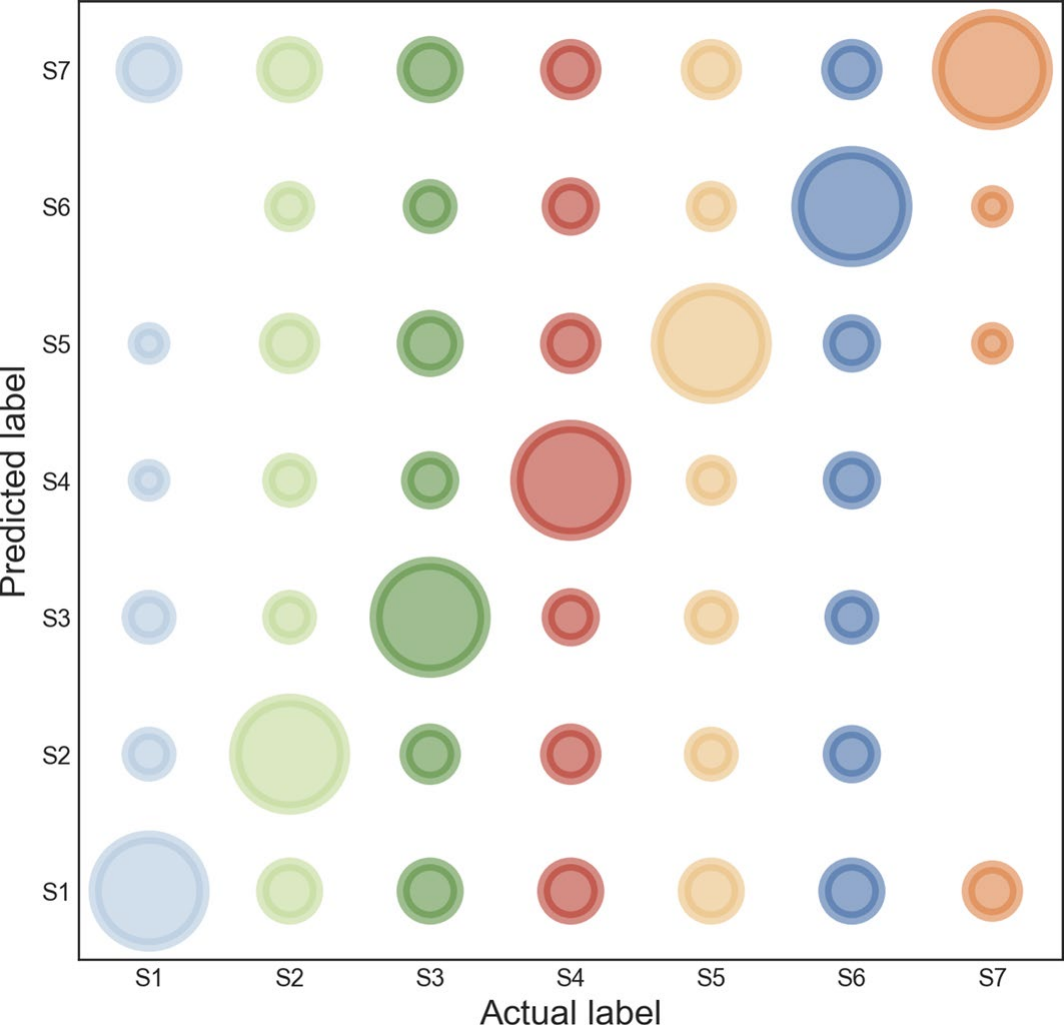
Real-predictive label-bubble chart in tenfold cross validation. The bubble sizes depict the predicted numbers of actual samples. (Python 3.8)

Compared with CGAN in Table 1, the indicators after the CWGAN were all improved, which demonstrated that the prediction performance of the generative network model was better after adding Wasserstein distance. Acc reached 0.8589; MCC was 0.8376; CEN and E_C_ were the smallest compared with other schemes, which suggested the strong ability of balancing data in CWGAN. Alternatively, we analyzed there might be similar sequence characteristics or structural features among divergent lysine modification types. Table 1 in Additional file: S3 and Fig. 2 showed the confusion matrix of the tenfold cross-validation results. Samples within S_1_ (Acetylation) were easily predicted to be S_7_ (Ubiquitination); samples within S_2_ (Glycation) were prone to classified into S_7_ and S_1_; some samples labelled as S_3_ (Malonylation) were wrongly predicted as the S_1_, S_7_ and S_5_ (Succinylation); samples labelled as S_4_ (Methylation) were specifically mis-predicted as S_1_; samples labelled as S_5_ were easily incorrectly predicted as S_1_; samples in S_6_ (SUMO) were mispredicted to S_1_ and S_7_; and S_7_ is easily mispredicted as S_1_. Therefore, acetylated sequences harbor the largest similarity with other modifications, indicating its function may be interconnected with other types. Sumoylation (S_6_) and ubiquitination (S_7_) were easily confused, further demonstrating the sequence or functional correlations between the two processes.

**Fig. 3:**
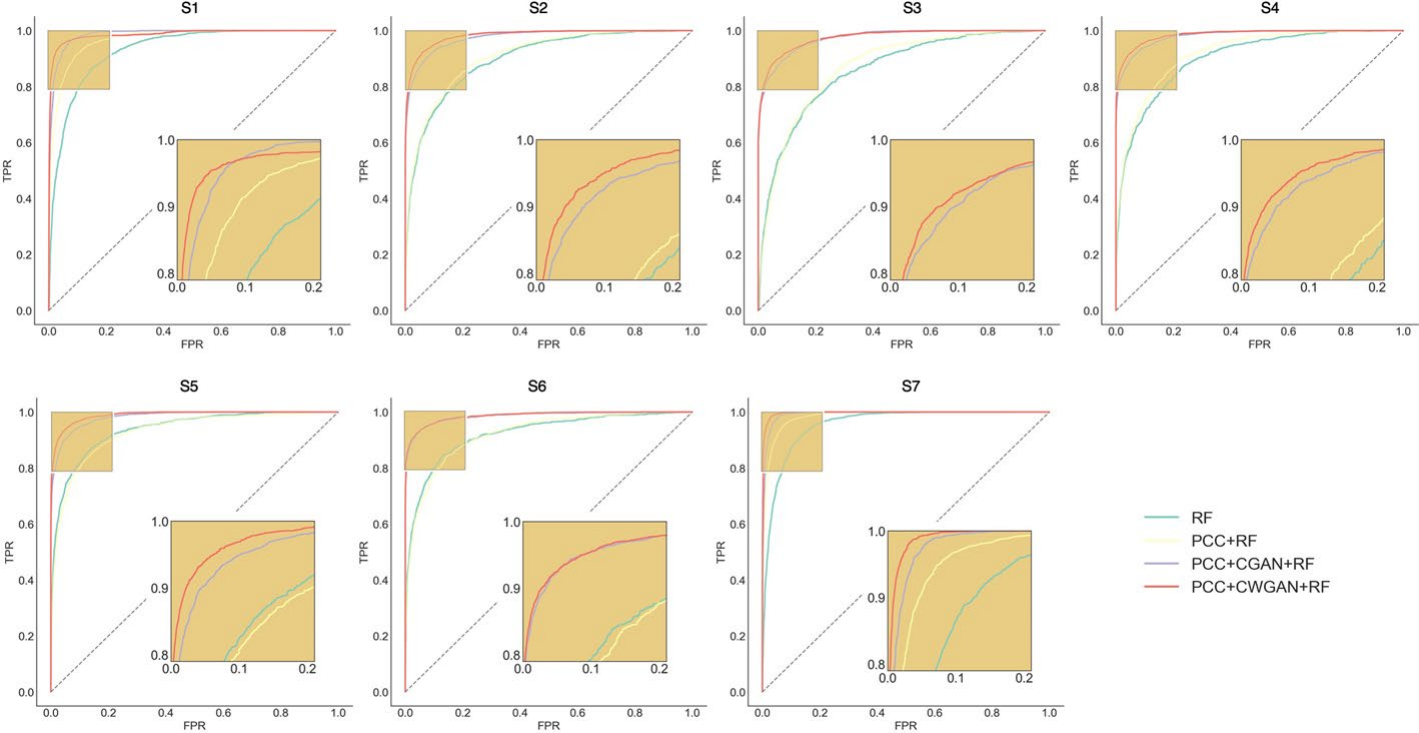
ROC curves of seven modification types with random forest in the tenfold cross-validation after PCC, CGAN and CWGAN. True positive rate (sensitivity) as the ordinate, false positive rate as the abscissa. The performance of random forest (RF) classification (baseline), RF with PCC screening, RF with PCC screening and CGAN augmentation, RF with PCC screening and CWGAN augmentation are visualized by green, yellow, purple and red, respectively. Acetylation (S1), Glycation (S2), Malonylation (S3), Methylation (S4), Succinylation (S5), Sumoylation (S6) and Ubiquitination (S7). (Python 3.8)

Table 2 and 3 showed the prediction results in each category before and after CWGAN. Strikingly, after CWGAN, the values of Sn were significantly increased, and the false negative rate of prediction was largely reduced. For the balanced AUC value of each category, there was a substantial increase and CWGAN reflected best prediction performance. Figure 3 demonstrated the comprehensive performance of each modification using PCC, CGAN and CWGAN in the training data. To testify whether the prediction AUCs based on different methods are significant, we used DeLong test, one nonparametric test which can compare AUC of two correlated ROC curves. From Table 2 in Additional file: S3, we underscored that PCC + CWGAN + RF was significantly better than RF and PCC + RF, and for S_1_, S_2_, S_4_, and S_6_, PCC + CWGAN + RF performed significantly better than PCC + CGAN + RF. No statistically better performance in S_3_, S_5_, and S_7_ was shown compared PCC + CWGAN + RF with PCC + CGAN + RF.

**Table 2:** Evaluation of each modification in tenfold cross-validation before CWGAN

	Acc	Sp	Sn	MCC	AUC
*S*_*1*_	0.8510	0.8396	0.8867	0.6583	0.9682
*S*_*2*_	0.9169	0.9643	0.5327	0.5421	0.9080
*S*_*3*_	0.9177	0.9827	0.2891	0.3912	0.8693
*S*_*4*_	0.9362	0.9857	0.4372	0.5435	0.9185
*S*_*5*_	0.9140	0.9545	0.6411	0.6096	0.9300
*S*_*6*_	0.9390	0.9885	0.4510	0.5723	0.9206
*S*_*7*_	0.9065	0.8981	0.9326	0.7749	0.9829

**Table 3:** Evaluation of each modification in tenfold cross-validation after CWGAN

	Acc	Sp	Sn	MCC	AUC
*S*_*1*_	0.9289	0.9347	0.8939	0.7485	0.9859
*S*_*2*_	0.9625	0.9851	0.8253	0.8408	0.9830
*S*_*3*_	0.9593	0.9912	0.7703	0.8274	0.9749
*S*_*4*_	0.9711	0.9937	0.8362	0.8786	0.9857
*S*_*5*_	0.9658	0.9811	0.8743	0.8602	0.9897
*S*_*6*_	0.9730	0.9957	0.8345	0.8848	0.9848
*S*_*7*_	0.9572	0.9539	0.9771	0.8507	0.9964

### Independent test results

**Fig. 4:**
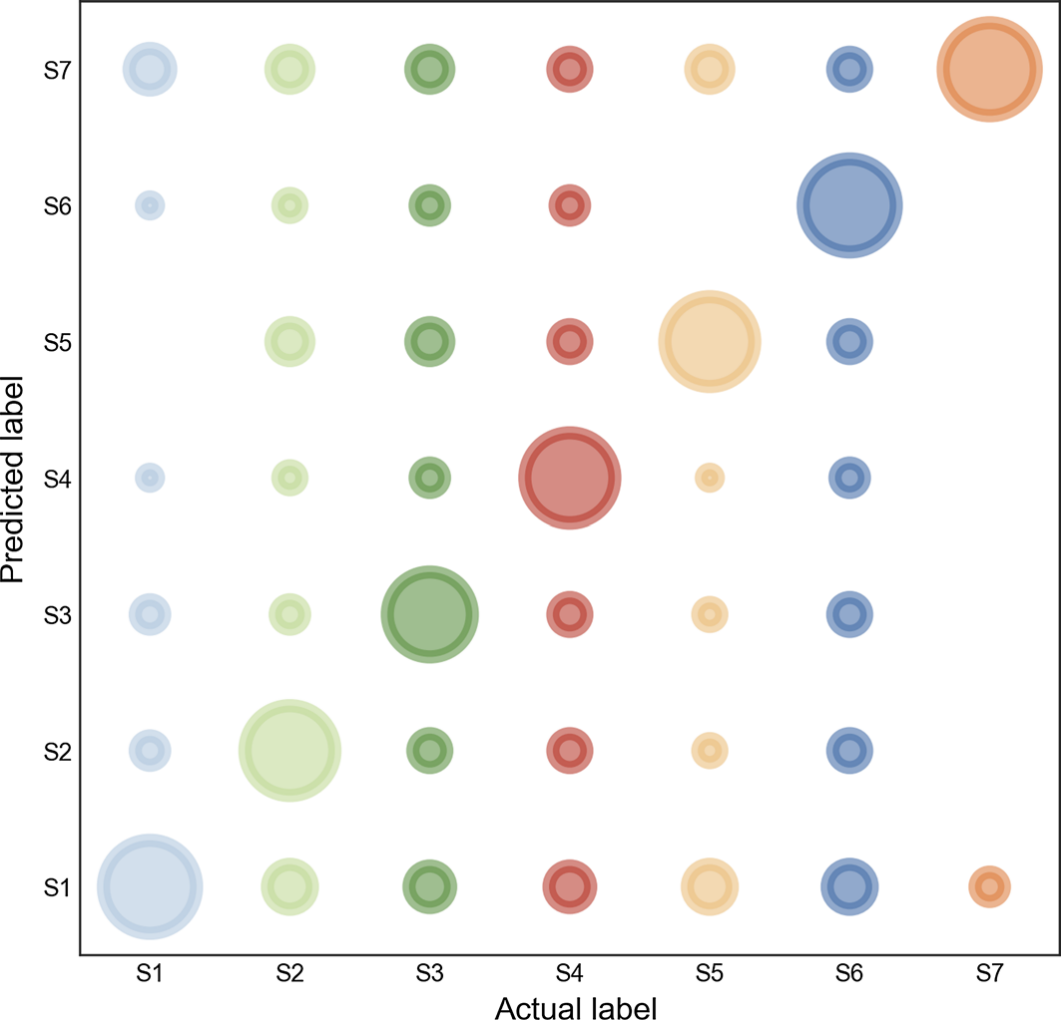
Real-predictive label bubble chart for independent test. The bubble sizes indicate the predicted numbers of actual samples. (Python 3.8)

**Fig. 5:**
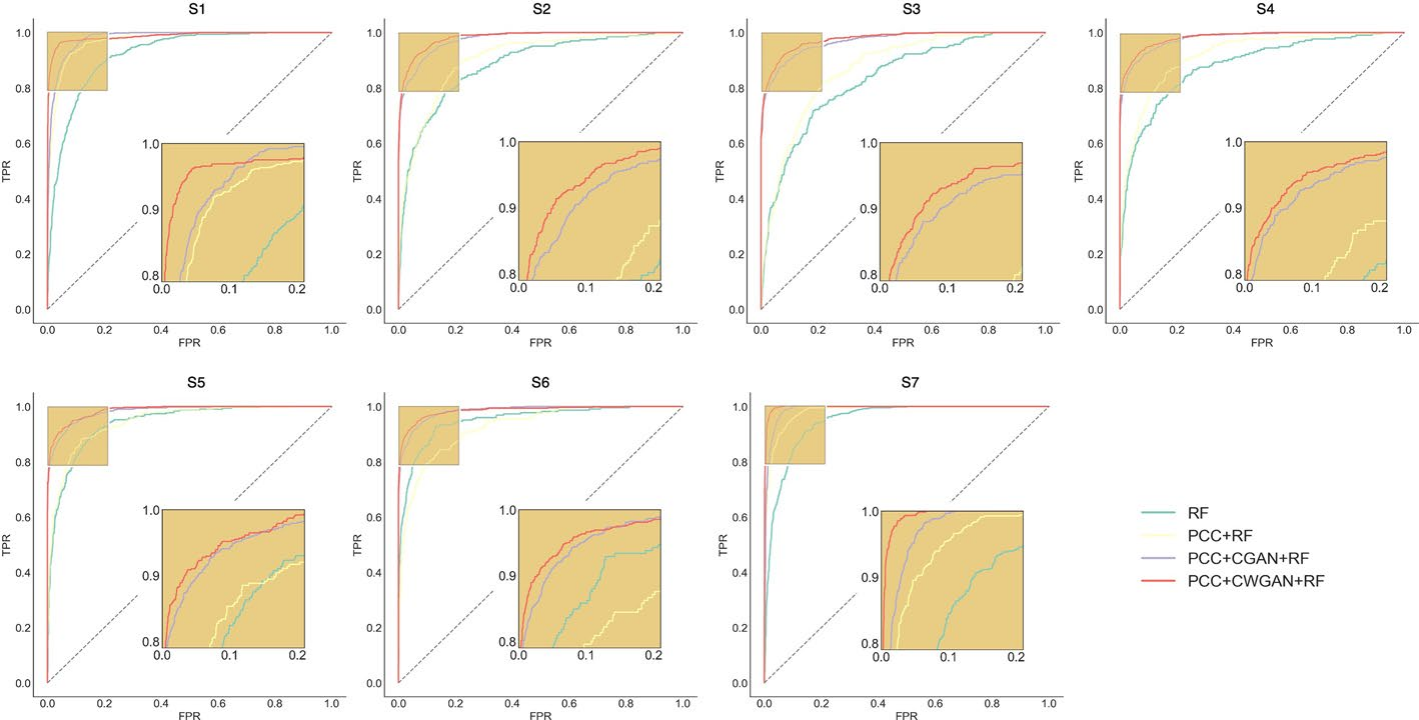
ROC curves of seven modification types in independent test. True positive rate (sensitivity) as the ordinate, false positive rate as the abscissa. The performance of random forest (RF) classification (baseline), RF with PCC screening, RF with PCC screening and CGAN augmentation, RF with PCC screening and CWGAN augmentation are visualized by green, yellow, purple and red, respectively. Acetylation (S1), Glycation (S2), Malonylation (S3), Methylation (S4), Succinylation (S5), Sumoylation (S6) and Ubiquitination (S7). (Python 3.8)

Profiling the independent dataset which is orthogonal to training set, the results in Table 4 were consistent with the training results (Table 1), which illustrated the robustness of our predictor. Additionally, the realistic and predicted lysine modification types elucidated the similar mechanisms constant with cross-validation results, which provided an effective way to inform the possibly functional connections among different types (Fig. 4, Table 3 in Additional file: S3). The value of Acc was 0.8549, MCC was 0.8330, CEN was 0.2250 and E_I_ was 0.1451 after CWGAN in the independent cohort. Table 5 and 6 demonstrated the better predictive performance after CWGAN for each modification type compared without CWGAN. Figure 5 enumerated ROC curves for each modification and the high AUCs suggested that MultiLyGAN harbored excellent predictive ability for the unseen data. In contract to only RF and RF without augmented data, there was a significant improvement in PCC + CWGAN + RF (Table 4 in Additional file: S3). For S_1_ and S_3_, PCC + CWGAN + RF harbored more precise prediction than PCC + CGAN + RF. The CWGAN and CGAN showed no discriminative difference in the remaining types.

**Table 4:** Comparisons of independent test results after PCC, CGAN and CWGAN

	Acc	MCC	CEN	E_I_
*Before PCC*	0.6391	0.5553	0.4185	0.3609
*After PCC*	0.6946	0.6251	0.3856	
*After CGAN*	0.8208	0.7937	0.2654	0.1792
*After CWGAN*	0.8549	0.8330	0.2250	0.1451

**Table 5:** Evaluation of each modification in independent test before CWGAN

	Acc	Sp	Sn	MCC	AUC
*S*_*1*_	0.8674	0.8563	0.9022	0.6924	0.9723
*S*_*2*_	0.9101	0.9598	0.4794	0.4780	0.9049
*S*_*3*_	0.9058	0.9759	0.2780	0.3513	0.8695
*S*_*4*_	0.9403	0.9856	0.4444	0.5445	0.9192
*S*_*5*_	0.9233	0.9603	0.6550	0.6312	0.9439
*S*_*6*_	0.9411	0.9864	0.4667	0.5701	0.9270
*S*_*7*_	0.9012	0.8939	0.9216	0.7689	0.9815

**Table 6:** Evaluation of each modification for independent test after CWGAN

	Acc	Sp	Sn	MCC	AUC
*S*_*1*_	0.9264	0.9314	0.8970	0.7443	0.9868
*S*_*2*_	0.9601	0.9863	0.8125	0.8386	0.9828
*S*_*3*_	0.9592	0.9865	0.7862	0.8193	0.9769
*S*_*4*_	0.9711	0.9945	0.8278	0.8761	0.9845
*S*_*5*_	0.9610	0.9776	0.8587	0.8375	0.9863
*S*_*6*_	0.9686	0.9955	0.8186	0.8741	0.9856
*S*_*7*_	0.9634	0.9592	0.9902	0.8672	0.9974

We investigated one case study on distinguishing two confusing modifications easily to be misclassified, which was illustrated in our paper. For example, Ubiquitination and Acetylation (Fig. 2) were reported they had a direct competition [42], and the opposing role between the two PLMs was successfully supported by mass spectrometric profiling [43]. Additionally, recent papers proposed that more complicated crosstalk mechanism was revealed referring to cell cycle regulation [44]. Therefore, the signaling pathways regulated by the two PLMs might affect the function of proteins, possibly leading to difficult identification. Therefore, it is essential to detect the true label. According to Fig. 1 of Additional file: S3, we showed the detailed mis-prediction results, and the thickness of each line was proportional to the number of misclassified samples. Aided by CWGAN, there was an apparent improvement on that fragments labelled as Ubiquitination were wrongly classified into Acetylation.

### Data augmentation results

**Fig. 6:**
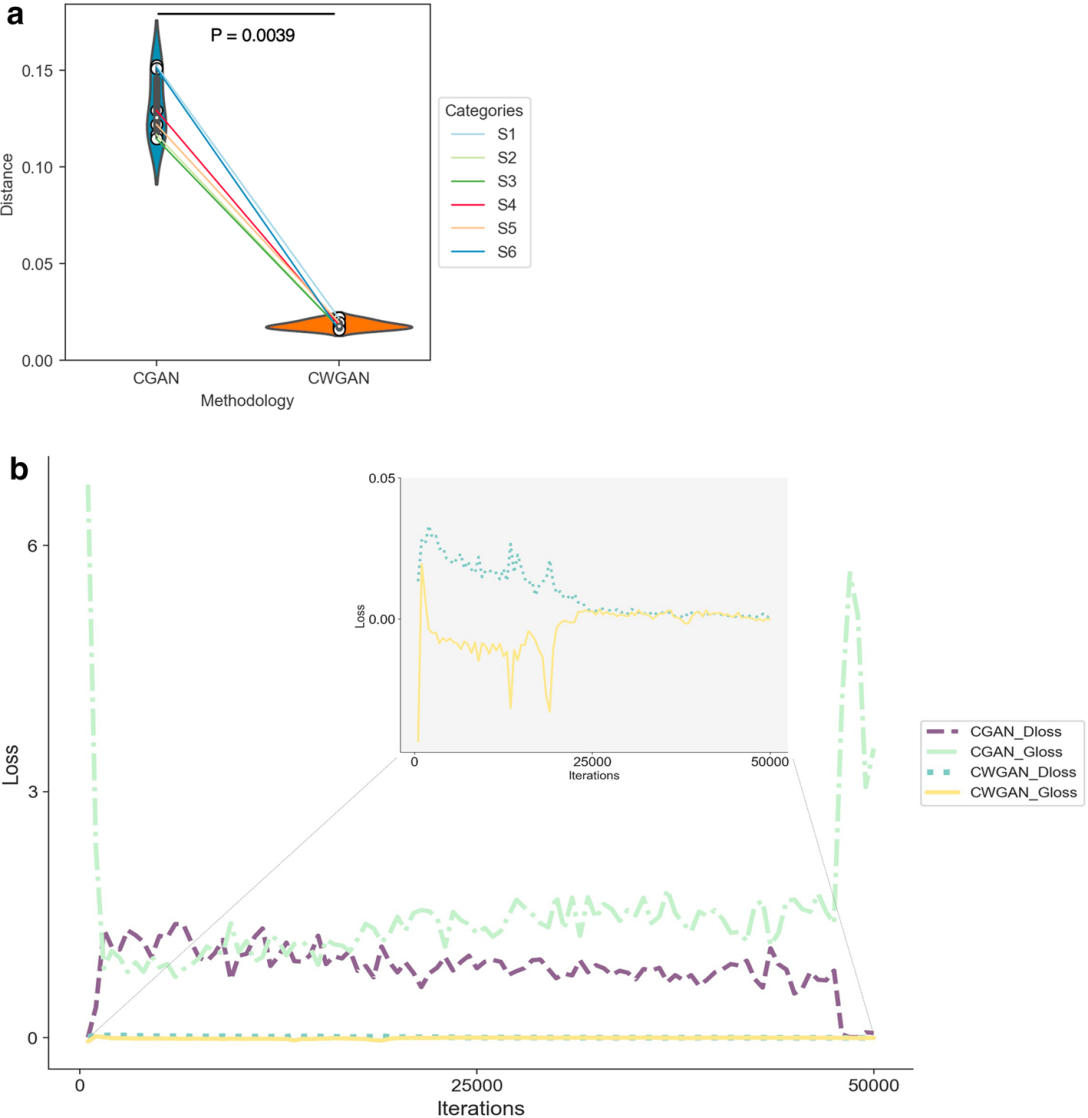
**a** Distance comparisons of 6 categories between CGAN and CWGAN. The Euclidean distance was calculated between the mean value of the simulated data and the real data. Distances using CGAN are all above 0.1, while distances with CWGAN of 6 categories are below 0.03, indicating that simulation data aided by CWGAN are more similar to the original real data. *P* value was calculated by two-sided Mann–Whitney *U* test. **b** Loss vs iterations graph of CGAN and CWGAN. The loss iteration graph reflects the stability of the iterative process of different algorithms, which proves that the CWGAN-training process is more even. (Python 3.8)

In addition to the improvement of predictive performance, we evaluated CGAN and CWGAN on loss variations in neural network training. This paper adopted the average distance to evaluate simulation data after GAN training. Firstly, the mean of all real data was calculated. Secondly, the Euclidean distance was calculated between the mean value of the simulated data and the real data for each category (modification) as the distance (Fig. 6a). CGAN's distances were all above 0.1, while CWGAN's distances of 6 categories were below 0.03, which indicated that CWGAN's synthetic data were more similar to the original real data. Distances of CGAN fluctuated, while CWGAN’s was stable in different categories.

We calculated the loss of the generator (Gloss) and the loss of the discriminator (Dloss) during 50,000 iterations to compare the advantages and disadvantages of the two algorithms. The Gloss and Dloss in training CGAN and CWGAN on the acetylation modification (S_1_) was depicted in Fig. 6b, where the upper subgraph is an enlarged graph of CWGAN’s loss coordinate. In the early iterations, the loss of Gloss and Dloss of CGAN tended to be highly changed within 500 iterations. However, it showed long-time fluctuations in the later iterations and eventually showed no convergence. In contrast, the Gloss and Dloss of CWGAN were relatively even and showed no longer changed after 25,000 iterations. Collectively, we confirmed that more stable and convergent augmented data can be accessed in CWGAN training process. Six other modifications had similar results as acetylation.

To testify whether CWGAN had superb performance in comparison to traditional oversampling methods, we applied the Synthetic Minority Oversampling Technique (SMOTE) to conduct the same steps for comparable results. The tenfold cross-validation and independent matrices of SMOTE were lower than CWGAN, and higher than no-augmentation (Table 7, 1 and 4), implying imbalanced data types literally led to worse results and CWGAN gave the more precise predictions compared with SMOTE.

**Table 7:** Performance of SMOTE in tenfold Cross-validation and independent test

	Acc	MCC	CEN	E_C_
Tenfold	0.8048	0.7730	0.2942	0.1952
Independent test	0.7948	0.7614	0.3044	0.2052

## Discussion

### Feature analysis

**Fig. 7:**
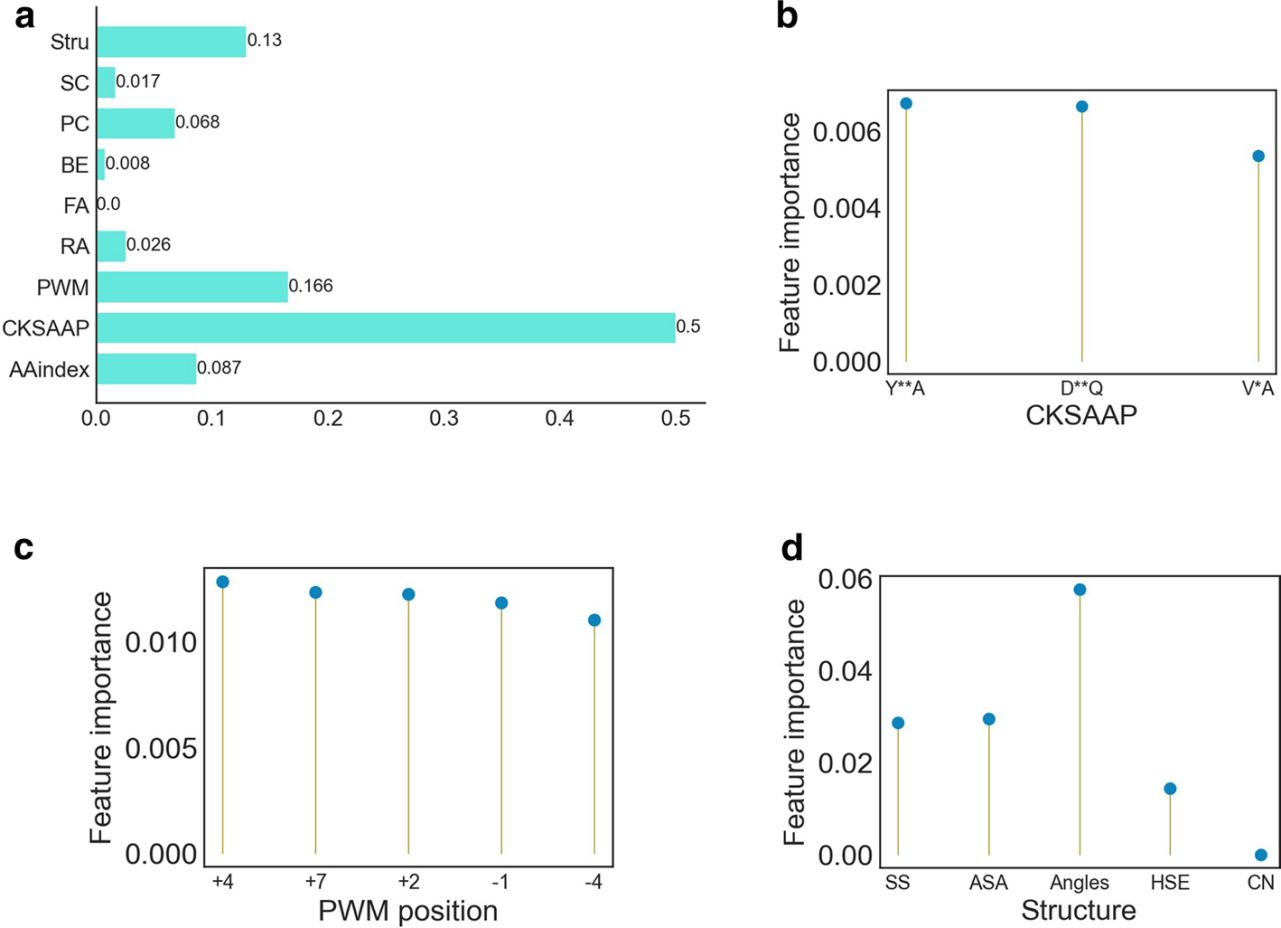
**a** Ranking chart of feature importance summarized by different feature-construction methods. **a** shows that CKSAAP, PWM and Structure coding are the three most informative methods; the importance of FoldAmyloid coding is 0, which provides no identification information. The remaining three images are the three most important amino-acid-space combination in CKSAAP **(b)**, the position information of the top five amino acids in PWM **(c)**, and the cumulative importance of the structural information of five signatures **(d)**

RF gave the order of importance for the 1497-dimensional features. According to the importance degree, the first nine most important features were from the PWM-encoding scheme. The frequency of different amino acids appearing in different positions of the sequence fragment was significantly different, which provided important information. The cumulative importance of different encoding schemes is summarized in Fig. 7. The importance of FoldAmyloid is 0, which provides no identification information; CKSAAP, PWM and structure features are the three most important indicators (Fig. 7a). Figure 7b shows the margin of the three most important amino acids in CKSAAP. Y**A, D**Q and V*A play key roles in the fragments, indicating that there is a significant difference. Figure 7c shows the position information of the top five amino acids in PWM. The amino acid frequency information at + 4, + 7, + 2, − 1 and − 4 positions also differs significantly during different categories. Figure 7d shows the cumulative importance of the structural information of five amino acids. CN showed no contribution, whereas angles showed the largest cumulative contribution. After analysis, it was found that the top three features in structure-encoding schemes were all from secondary structure (SS), indicating that the SS plays an important role in identification.

### Comparison with other existing methods

To validate the performance of MultiLyGAN, the comparison of our models with the MusiteDeep [45] was performed. MusiteDeep, a deep-learning based predictor, provided identification for multiple PTMs, including 13 PTMs of which five are lysine-based modifications (Comparisons of number of enrolled proteins and modification sites were in Table 5 of Additional file: S3). We tested four PLMs which are also discussed in MusiteDeep. Using the same independent dataset illustrated above, we analyzed their performance in Table 8. Our method outperformed the MusiteDeep for identification of all four types of PLMs. In MusiteDeep, the Sp in each modification type was obviously higher than Sn, suggesting the lower detection ability for true positive modification types, which was improved by our method.

**Table 8:** Comparisons of performance between MusiteDeep with MultiLyGAN

	PLMs	Acc	Sp	Sn	MCC	AUC
MusiteDeep	Ubiquitination	0.6641	0.8078	0.2261	0.0365	0.5255
	Sumoylation	0.6668	0.6863	0.4723	0.0972	0.6125
	Acetylation	0.5039	0.4818	0.5730	0.0471	0.5512
	Methylation	0.8386	0.9093	0.1135	0.0224	0.4773
MultiLyGAN Our method	Ubiquitination	0.9634	0.9592	0.9902	0.8672	0.9974
	Sumoylation	0.9686	0.9955	0.8186	0.8741	0.9856
	Acetylation	0.9264	0.9314	0.8970	0.7443	0.9868
	Methylation	0.9711	0.9945	0.8278	0.8761	0.9845

## Conclusions

In this work, we propose a new pipeline to predict the seven types of modified sites, where the GAN was utilized to solve the data imbalance problem. We translated the multilabel prediction problem into a multiclass prediction problem. Overall, 2340 dimensional features were constructed by combining eight different sequences and five structural information-encoding schemes. Finally, 1497 dimensional features were obtained after PCC feature extraction. Through CWGAN, the generated simulation data were closer to the real data. CWGAN yielded Acc of 0.8549, MCC of 0.8330, CEN of 0.2250, and E_I_ of 0.1451 by independent test, which were better scores than those obtained by CGAN. Meanwhile, CWGAN performed better in each of the seven modifications than CGAN.

## Methods

### Method overview

As illustrated in Fig. 1a, we proposed an integrated protocol including data preprocessing, feature construction, dimensionality reduction, sample augmentation and classification, which implemented stratifications of seven lysine modification types. Preparation of peptide fragments, followed by discarding homologous sequences, was finished in data preprocessing module. Subsequently, substantial sequential and structural signatures were exacted for each sample in feature construction module, after which we used Pearson correlation coefficient (PCC) to acquire principal features in a lower dimensional subspace. To minimize the influence of imbalanced problem that the minority class is prone to incorrectly classified, Conditional Generative Adversarial Network (CGAN) and Conditional Wasserstein Generative Adversarial Network (CWGAN) were carried out. Finally, we built Random Forest (RF) classifiers to identity the seven subtypes, and model performance of multiclass classification was measured by Accuracy (Acc), Confusion Entropy (CEN), Matthews Correlation Coefficient (MCC), Cross-validation error rate (E_C_) and independent test error rate (E_I_) (Details are shown in Additional file: S4). MultiLyGAN consisted of PCC, CWGAN and RF.

### Data preprocessing

We collected 18 kinds of lysine modification samples from the CPLM2.0 database [46], involving a total of 284,780 modification sites from 53,501 proteins. The types of modifications were Ubiquitination, Acetylation, Succinylation, Malonylation, Sumoylation, Glycation, Methylation, Glutarylation, Propionylation, Crotonylation, Pupylation, Butyrylation, Formylation, Phosphoglycerylation, Hydroxylation, 2-hydroxyisobutyrylation, Neddylation, and Carboxylation. Peptide fragments were obtained through a sliding window technique, with length ξ = 8 in the upper and lower lysine amino acid (window size *L* = 17). To reduce redundancy and bias, fragments with high sequence similarity (40% or more pairwise sequence identity) were removed. After deleting homology, we obtained 46 2-hydroxyisobutyrylated, 3273 acetylated, 38 butyrylated, 16 carboxylated, 29 crotonylated, 143 formylated, 402 glutarylatd, 1454 glycated, 19 hydroxylated, 1467 malonylated, 1208 methylated, 37 neddylated, 108 phosphoglycerylated, 223 propionylated, 169 pupylated, 1855 succinylated, 1302 sumoylated and 3468 ubiquitinated lysine-centered fragments. These data totally contained 18 different modification types.

We merged the data with the same ID, site and fragments. Eighteen types of modifications contribute to theoretically 2^18^ types of labels for each fragment. After data integration, there were a total of 58 schemes, encompassing 18 for one label, 28 for two labels, and 12 for three labels. The labels with less than 500 samples were deleted. The remaining samples are single-label data, including Ubiq (3253), Ace (3194), Succ (1692), Glyca (1416), Malon (1253), Sumo (1213) and Meth (1172). Since the feature construction consisted of the structural information of each amino acid, we discarded fragments with the length less than 17. Finally, we attained Ubiq (3185), Ace (3114), Succ (1645), Glyca (1399), Malon (1224), Sumo (1174) and Meth (1147). The detailed data of each type are shown in Fig. 1b and Table 6 in Additional file: S3.

In alphabetical order, S_1_ was set as Ace, S_2_ as Glyca, S_3_ as Malon, S_4_ as Meth, S_5_ as Succ, S_6_ as Sumo, and S_7_ as Ubiq. Thus, the total dataset S can be defined as:

1 $$S={S}_{1}\cup {S}_{2}\cup {S}_{3}\cup {S}_{4}{\cup S}_{5}\cup {S}_{6}\cup {S}_{7}$$

## Feature construction

### Sequence feature

#### AAindex [47, 48]

Fourteen specific physical and chemical properties were selected to construct features. A 14-dimensional vector was obtained for every amino acid (*L* is the length of the fragment):

2 $$\left( {f\left( 1 \right),{ }f\left( 2 \right), \ldots \ldots ,{ }f\left( {14L} \right)} \right).$$

#### CKSAAP [49]

The margin K is 0, 1, and 2 between the amino acid pair. If the pair was AA, CKSAAP is “AA,” “AXA,” and “AXXA,” where X is any amino acid. The number 1, 2, 3, …, denotes amino acids according to alphabetical order A, C, D, …, Y. The sample is encoded as:

3 $$\left(f\left(\mathrm{1,0},1\right),\dots ,f\left(\mathrm{20,0},20\right),f\left(\mathrm{1,1},1\right),\dots ,f\left(\mathrm{20,1},20\right),f\left(\mathrm{1,2},1\right),\dots ,f\left(\mathrm{20,2},20\right)\right).$$

There are 400 dimensions for each margin *k* (0, 1, and 2) value, and a 1200-dimensional vector was obtained.

#### PWM [50, 51]

The position weight matrix was calculated by category to obtain the frequency information of the amino acids at each position. According to the above description, the total length of the sample fragment is *L*, thus, each sample can be encoded as *L*-dimensional vector:

$$\left(f\left(1\right), f\left(2\right),\dots \dots , f\left(L\right)\right)$$. (4).

#### Reduced Alphabet [52, 53]

A reduced-letter code 8 is selected, and each amino acid is encoded as an 8-dimensional vector by acid, basic, aromatic, amide, small hydroxyl, sulfur, aliphatic 1 and aliphatic 2. Therefore, a sample of length L is encoded as a vector of 8 × *L*:

5 $$\left(f\left(1\right), f\left(2\right),\dots \dots , f\left(8L\right)\right).$$

#### FoldAmyloid [54]

Using http://antares.protres.ru/fold-amyloid/ to predict the amyloidogenic region of the sample and finally obtain the *L*-dimensional vector:

$$\left(f\left(1\right), f\left(2\right),\dots \dots , f\left(L\right)\right)$$. (6).

#### BE [5, 55]

Under BE (Binary Encoding), each amino acid is encoded into a 20-dimensional binary vector, resulting in a 20 × *L*-dimensional vector:

7 $$\left(f\left(1\right), f\left(2\right),\dots \dots , f\left(20L\right)\right)$$

#### PC-PseAAC [56, 57]

Select λ = *L*-1 = 17–1 = 16, ω = 0.05, physicochemical properties = [‘Hydrophilic’, ‘Hydrophobic’, ‘Quality’]. Each peptide fragment ultimately obtained a (20 + *L*-1)-dimensional vector:

8 $$\left(f\left(1\right), f\left(2\right),\dots , f\left(20\right),f(20+1)\dots ,f(20+L-1)\right) .$$

#### SC-PseAAC [57, 58]

Select λ = *L*-1 = 17–1 = 16, ω = 0.05, physicochemical properties = [‘Hydrophilic’, ‘Hydrophobic’]. Each protein fragment ultimately obtained the characteristics of a (20 + 2(*L*-1))-dimensional vector:

9 $$\left(f\left(1\right), f\left(2\right),\dots , f\left(20\right),f(20+1)\dots ,f(20+2(L-1))\right).$$

## Structure feature

SPIDER3-Single [59] applies LSTM-BRNN to predict Accessible Surface Area (ASA), secondary structure (SS), backbone torsion angles (φ, ψ, θ, τ), Half Sphere Exposure (HSE) and Contact Number (CN), which had a total of 19 outputs. The first was ASA; the next 3 nodes (SS, Q3) were helix (H), strand (E) and coil (C); the next 8 (SS, Q8) were 3_10_-helix (G), α-helix (H), $\pi$-helix (I), $\beta$-bridge (B), $\beta$-strand (E), $\beta$-turn (T), bend (S) and coil(C); the next 4 were φ, ψ, θ, and τ; the next 2 were HSEα-up and HSEα-down, and the last output code was CN. SS yields an 11-dimensional vector; ASA is 1-D; φ, ψ, θ, τ are 4-D; HSE is 2-D (HSEα-up, HSEα-down); and CN is 1-D. Therefore, we collected a 19 *L*-dimensional vector for each protein fragment. Combining sequence and structural features, each peptide was translated into a 2359-dimensional vector (see Table 7 in Additional file: S3).

### Sample augmentation

#### CGAN

GAN has shown its excellent performance in training a generative model. However, there is no control on generative models in GAN and the data being generated are completely random without any information of categories, making it impossible to deal with the imbalance issue. Fortunately, the CGAN model was proposed to direct the data generation process by conditioning the model on additional information, such as class labels. An easy way to extend a GAN to a conditional model is conditioning both the generator and discriminator on some extra information y. The optimization function of CGAN as follow:

10 $$L=\underset{D}{\mathrm{max}}({E}_{x\sim {P}_{data}(x)}\left[logD\left(x|y\right)\right]+{E}_{z\sim {P}_{G}\left(z\right)}[\mathrm{log}(1-D(G\left(z|y\right)))])$$

#### CWGAN

We used CWGAN (Conditional Wasserstein Generative Adversarial Network, CGAN under Wasserstein’s method) model, which integrates CGAN and Wasserstein’s distance. The objective of GAN is to learn best parameters for generator so as to minimize the JS divergence between the real distribution ${P}_{data}\left(x\right)$ and the simulated distribution ${P}_{G}\left(x\right)$. However, these two distributions usually have no overlap in sample space, which make their JS divergence always equal to log2 and lead to 0 gradient for parameters of generator. It is difficult for GAN to improve the performance of generator because of the 0 gradient. Therefore, a better method has been proposed to measure the divergence between distributions, which is called as ﻿Wasserstein’s distance. When Wasserstein’s distance was used in Conditional GAN, CWGAN can start training even if ${P}_{data}\left(x\right)$ and ${P}_{G}\left(x\right)$ have no intersection. The optimization function of CWGAN as follow:

11 $$L=\underset{D}{\mathrm{max}}({E}_{x\sim {P}_{data}(x)}D\left(x|y\right)-{E}_{z\sim {P}_{G}\left(z\right)}D(G\left(z|y\right))$$

For the generator, the input includes the prior noise distribution z and the categorical label y embedded as a seven-dimensional vector by one-hot encoding method. There are several main improvements in the network. CWGAN deleted the sigmoid function of the last layer of D. The loss function for G and D no longer used logarithmic transformation. Instead, it used the clip function to update the function and replace Adam with the RMSProp optimization method. Different from common WGAN that outputs the divergence of generative samples in comparison to realistic samples, the discriminator in CWGAN further adds the estimation of whether generative samples are matched to the conditional information. Therefore, CWGAN can generate samples with a specific category.

Using the sample amount of the seventh type of data as a reference, the CWGAN simulation was performed on the other six types, and the simulation data were consistent with the seventh type of data generated. The parameters tuning of CWGAN and the finial parameters of CWGAN are shown in Table 8 and Table 9 of Additional file: S3. Our training data for CWGAN was integrated samples with seven types after PCC screening, including 12,888 samples with 1497 features. After CWGAN, 71 first-class, 1786s-class, 1961 third-class, 2038 fourth-class, 1540 fifth-class, and 2011 sixth-class simulation data were generated. In total, 9407 simulated sample datasets were generated.

Random forest (RF) is a prevalent bagging approach of machine learning. The parameters of RF are shown in Table 10 of Additional file: S3.

### Measurements of performance

Two-classification and Multiclassification system indicators are in Additional file: S4.

## Supplementary Information

**Additional file 1. S1**: Sequence preprocessing. The supplementary material introduces amino acid window sliding technology and feature construction that convert amino acid sequences into numerical vectors.

**Additional file 2. S2**: CWGAN generative model.

**Additional file 3. S3**: Supplementary tables.

**Additional file 4. S4**: Classification system indicators.

## Data Availability

18 kinds of lysine modification samples were retrieved from the CPLM2.0 database [46] built by authors Dr. Zexian Liu and Dr. Yu Xue (http://cplm.biocuckoo.org/).

## Abbreviations

PTM: Protein post-translational modification
GAN: Generative adversarial network
CGAN: Conditional generative adversarial network
CWGAN: Conditional wasserstein generative adversarial network
Acc: Accuracy
CEN: Confusion entropy
MCC: Matthews correlation coefficient
RF: Random forest
E_I_: Independent test error rate
E_C_: Cross-validation error rate
PCC: Pearson correlation coefficient
