# Supplementary material for: Prediction and analysis of multiple protein lysine modified sites based on conditional wasserstein generative adversarial networks
Source: BMC Bioinformatics. 2021 Mar 31;22:171. doi: 10.1186/s12859-021-04101-y (PMC8010967; doi:10.1186/s12859-021-04101-y)
Supplement: Supplementary file 1 — Additional file 1. S1: Sequence preprocessing. The supplementary material introduces amino acid window sliding technology and feature construction that convert amino acid sequences into numerical vectors. [file 12859_2021_4101_MOESM1_ESM.docx]

**Sequence preprocessing**

## Amino acid window sliding technology

The 20 amino acids are combined into a protein having structural information by a process of dehydration condensation. Proteins with different structural information perform different functions. The primary sequence structure of a protein is determined by the order of amino acids constituting the sequence, and the order of amino acids is affected by the order in which the genetic code is arranged. In addition to the primary structural features of proteins carrying large amounts of information, secondary and tertiary structures also play a key role in the final structure of the protein and are related to the primary structure. It can be seen that the sequence characteristics of proteins are the most basic characteristics of proteins. Therefore, in the field of post-translational modification of protein prediction, how to select and construct accurate and reasonable numerical matrices based on amino acid sequence is the key to subsequent analysis.

The amino acid sliding window technique uses a specific amino acid as a sliding unit for sliding screening. It can locate all the central amino acids and capture the sequence fragments of the set window length, as shown in the following figure:

1 2 3

S A K W K S Y N Y M A C K Y

Figure 1 Sliding window to intercept amino acid sequence fragments

In Figure 1, the string represents a sequence of amino acids of length 14 and the red box represents the window being slid. Suppose the central amino acid is lysine, represented by the letter "K". Suppose the length of the sliding window is 7, and it is required to take the central amino acid as the center point, and take 3 amino acids in the upstream and downstream. There are 3 K in the figure, so the total sequence is cut into 3 amino acid fragments of length 7, those are XSAKMKS", "AKWKSYN" and "MACKYXX", in which the length less than 7 is complemented by the letter "X". Different segments are different samples.

## Feature construction

The process of converting an amino acid sequence into a numerical vector is called a feature construction. Amino acid sequences can be encoded in a variety of ways, such as: physicochemical properties (AAindex), K-space amino acid pair composition (CKSAAP), position-specific amino acid tendency (PSAAP), and position-specific propensity matrix (PSPM). Each amino acid can be represented by a capital letter, the specific representation is as follows: alanine (A), arginine (R), aspartic acid (D), cysteine (C), valley aminoamide (Q), glutamic acid (E), histidine (H), isoleucine (I), glycine (G), asparagine (N), leucine (L), lysine (K), methionine (M), phenylalanine (F), valine (P), serine (S), tryptophan (W), tyrosine (Y), valine ( V). A protein sequence can be represented using twenty amino acids plus a missing amino acid (X).

The eigenvector corresponding to the peptide P can be represented by the following formula:

$\mathbf{P}={(\psi_{1} \psi_{2}\cdots\psi_{u}\cdots\psi_{\Omega})}^{T}$ (1)$P={(\psi_{1} \psi_{2}\cdots\psi_{u}\cdots\psi_{\Omega})}^{T}$

For convenience, the peptide is defined as:

$\mathbf{P}=R_{1}R_{2}\cdots\cdots R_{L-1}R_{L}$ (2)

Where $R_{i}(i=1,2,\cdots,20;i\neq(L+1)/2,R_{(L+1)/2}=K)$ is any of the 20 natural amino acids.

**Amino Acid Index (AAindex)**

AAindex[1, 2] is a digital index database of various physicochemical and biochemical properties of amino acid and amino acid pairs. AAindex consists of three parts: AAindex1 is a list of 20 amino acid indices, AAindex2 is an amino acid substitution matrix, and AAindex3 is a list of contact potential indices for amino acid pairs. The amino acid database contains 566 indicators for describing the physical and chemical properties of each amino acid. This paper selects 14 indicators, so the AAindex encodes a protein with a vector of 14×L (which is the length of the amino acid sequence). The 14 indicators are hydrophobic, polarity, polarizability, solvent, accessibility, net charge index of side chains, molecular weight, PK-N, PK-C, melting point, optical rotation, entropy of formation, heat capacity and absolute entropy.

**Composition of K-space amino acid pairs (CKSAAP)**

CKSAAP[3, 4] encodes each protein sample into a 400-dimensional vector as follows: $(f\left( 1,k,1 \right),f\left( 1,k,2 \right),\cdots,f\left( 1,k,20 \right),f\left( r,k,s \right),\cdots f\left( 20,k,1 \right),\cdots,f(20,k,20))$. Where $(r,s=1,2,\cdots,20)$, $f(r,k,s)$ represents the amount of $k$ amino acids between the $r$-th amino acid and the $s$-th amino acid in the protein sample. Define the amino acid pair as $A_{i}A_{j}(i,j=1,2,\cdots,20)$. The frequency $f_{i,j}$ of all possible amino acid pairs is defined as:

$f_{i,j}=\frac{{Num(A}_{i}A_{j})}{L-K-1} (i,j=1,2,\cdots,20)$(3)

**Position weight matrix (PWM)**

PWM[5, 6] describes the frequency at which different amino acids appear at different positions in a sequence fragment, and is obtained by counting the frequency of 20 amino acids at different positions in all amino acid sequence fragments in the training set. Suppose the sequence length is L. If a row is used to represent an amino acid, the column indicates the position of the amino acid in the sequence fragment. Then the value of the *i-th* row and the *j-th* column of the PWM matrix indicates the frequency at which the *i-th* amino acid appears at the *j-th* position of the sequence fragment, where $i=1,2,\ldots,21,j=1,2,．．,w$. The sequence segment is encoded by an L-dimensional vector, and each dimension is the frequency at which the amino acid in the sequence appears at the corresponding position, that is, the corresponding value of the amino acid in the corresponding position of the PWM matrix.

**Reduced Alphabet**

According to the physicochemical properties of amino acids, biologists divide 20 amino acids into several groups[7, 8]. Different physicochemical properties are used for different groups. Reduced Alphabet is encoded according to amino acid grouping, and each amino acid encoding vector has the same dimensions as the number of groups. It is encoded by a binary vector, and Table 1 is the four grouping methods and encoding results.

Table Reduced alphabetic code list

| **The number of groups** | **Type** | **Amnio acid** | **Code** |
| --- | --- | --- | --- |
| three | Polar  Neutral  hydrophobic | RKEDQN  GASTPHY  CVLIMFW | 001  010  100 |
| four | Acidic  Basic  Polar  Nonpolar | DE  HKR  CGNQSTY  AFILMPVW | 0001  0010  0100  1000 |
| seven | Acidic  Basic  Aromatic  Amide  Small hydroxyl  Sulfur-containing  Aliphatic | DE  HKR  FYW  NQ  ST  CM  AGPILV | 0000001  0000010  0000100  0001000  0010000  0100000  1000000 |
| eight | Acidic  Basic  Aromatic  Amide  Small hydroxyl  Sulfur-containing  Aliphatic 1  Aliphatic 2 | ED  HKR  FYW  NQ  ST  CM  AGP  ILV | 00000001  00000010  00000100  00001000  00010000  00100000  01000000  10000000 |

**Amyloidogenic region (FoldAmyloid)**

The amyloidogenic region of the polypeptide chain is important because these regions are responsible for the formation and aggregation of amyloid. Therefore, it is useful to predict the location of the amyloidogenic region in the protein chain. FoldAmyloid[9] is a method for predicting amyloidogenic regions from protein sequences, introducing two features (expected probability of hydrogen bond formation and expected packing density of residues) to detect amyloidogenic regions in protein sequences. The experimental results show that the region with high skeleton-main chain hydrogen bond formation expected probability and the region with high expected bulk density are the main reasons for amyloid fibril formation. FoldAmyloid has been tested on a data set of 407 peptides and has shown good performance in predicting peptide status. The FoldAmyloid server is available at http://antares.protres.ru/fold-amyloid/.

**Binary code (BE)**

Binary coding is the most basic sequence coding method[10, 11]. In this paper, each of the 21 different amino acids (20 amino acids plus "X") is coded as a 21-dimensional vector containing only 0 and 1. Arrange amino acids in the order of ARNDCQEGHILKMFPSTWYVX.Amino acid A is represented by (1,0,0,0,0,0,0,0,0,0,0,0,0,0,0,0,0,0,0,0,0), and R is represented (0,1,0,0,0,0,0,0,0,0,0,0,0,0,0,0,0,0,0,0,0). Therefore, the dimension of the binary coded vector of each sample is 21 × L.

**Parallel related pseudo amino acid composition (PC-PseAAC)**

PC-PseAAC[12, 13] is a method of merging continuous local sequence order information and global sequence order information into feature vectors of protein sequences. Given a protein sequence P (formula 2), define the PC-PseAAC feature vector for P:

${\mathbf{P}={[ p}_{1} p_{2} \cdots p_{20} p_{20+1} {\cdots p}_{20+\lambda} ]}^{T}$ (4)

Among them:

$p_{u}=\left\{ \begin{aligned} \begin{matrix} \frac{f_{u}}{\sum_{i=1}^{20} f_{i}+w\sum_{j=1}^{\lambda} \theta_{j}}, & 1\leq u\leq20 \end{matrix} \\ \begin{matrix} \frac{w\tau_{u}}{\sum_{i=1}^{20} f_{i}+w\sum_{j=1}^{\lambda} \theta_{j}}, & 20+1\leq u\leq20+\lambda\end{matrix} \end{aligned} \right.$(5)

Where $f_{i}\left( i=1,2,3,\cdots,20 \right)$ is the normalized frequency of occurrence of 20 natural amino acids in protein P. The parameter λ is an integer representing the highest count of correlations along the protein sequence; w is a weighting factor ranging from 0 to 1; $\theta_{j}\left( i=1,2,3,\cdots,\lambda\right)$ is called the j-layer correlation factor, reflecting the sequence order correlation between all j-th most contiguous residues along the protein chain, which is defined as follows:

$\left\{ \begin{aligned} \begin{matrix} \theta_{1}=\frac{1}{L-1}\sum_{i=1}^{L-1} \Phi(R_{i},R_{i+1}) \\ \theta_{2}=\frac{1}{L-2}\sum_{i=1}^{L-2} \Phi(R_{i},R_{i+2}) \end{matrix} \\ \begin{matrix} \theta_{3}=\frac{1}{L-3}\sum_{i=1}^{L-3} \Phi(R_{i},R_{i+3}) \\ \cdots\\ \theta_{\lambda}=\frac{1}{L-\lambda}\sum_{i=1}^{L-\lambda} \Phi(R_{i},R_{i+\lambda}) \end{matrix} \end{aligned} \right., (\lambda<L)$ (6)

Where the correlation function is given by:

$$\Phi\left( R_{i},R_{j} \right)=\frac{1}{3}\left\{ \left[ H_{1}\left( R_{j} \right)-H_{1}\left( R_{i} \right) \right]^{2}+\left[ H_{2}\left( R_{j} \right)-H_{2}\left( R_{i} \right) \right]^{2}+\left[ M\left( R_{j} \right)-M\left( R_{i} \right) \right]^{2} \right\}$$

(7)

Where $H_{1}\left( R_{i} \right)$, $H_{2}\left( R_{i} \right)$ and $M\left( R_{i} \right)$ are the hydrophobicity value, hydrophilicity value and side chain mass. Before substituting the values of hydrophobicity, hydrophilicity and side chain mass into Formula 7, they all need to be standardized, as described below:

${\begin{matrix} H_{1}\left( R_{i} \right)=\frac{H_{1}^{0}\left( R_{i} \right)-\sum_{k=1}^{20} \frac{H_{1}^{0}\left( \Theta_{k} \right)}{20}}{\sqrt{\sum_{u=1}^{20} \frac{{{[H}_{1}^{0}\left( R_{u} \right)-\sum_{k=1}^{20} \frac{H_{1}^{0}\left( \Theta_{k} \right)}{20}]}^{2}}{20}}} \\ H_{2}\left( R_{i} \right)=\frac{H_{2}^{0}\left( R_{i} \right)-\sum_{k=1}^{20} \frac{H_{2}^{0}\left( \Theta_{k} \right)}{20}}{\sqrt{\sum_{u=1}^{20} \frac{{{[H}_{2}^{0}\left( R_{u} \right)-\sum_{k=1}^{20} \frac{H_{2}^{0}\left( \Theta_{k} \right)}{20}]}^{2}}{20}}} \end{matrix} \atop M\left( R_{i} \right)=\frac{M^{0}\left( R_{i} \right)-\sum_{k=1}^{20} \frac{M^{0}\left( \Theta_{k} \right)}{20}}{\sqrt{\sum_{u=1}^{20} \frac{{{[M}^{0}\left( R_{u} \right)-\sum_{k=1}^{20} \frac{M^{0}\left( \Theta_{k} \right)}{20}]}^{2}}{20}}}}$(8)

Where $H_{1}^{0}\left( R_{i} \right)$ is the original hydrophobicity value of the *i-th* amino acid, $H_{2}^{0}\left( R_{i} \right)$ is the corresponding original hydrophilicity value, and $M^{0}\left( R_{i} \right)$ is the *i-th* amino acid side chain mass. $\Theta_{i} \left( i=1,2,3,\cdots,20 \right)$ represents 20 natural amino acids according to the alphabetical order of their one-letter codes: A, C, D, E, F, G, H, I, K, L, M, N, P, Q, R, S, T, V, W and Y.

**Sequence-related pseudo amino acid composition (SC-PseAAC)**

SC-PseAAC is a variant of PC-PseAAC. Given a protein sequence P (Formula 2), define the SC-PseAAC eigenvector of P:

${\mathbf{P}={[ p}_{1} p_{2} \cdots p_{20} p_{20+1}{\cdots p_{20+\lambda} p}_{20+\lambda+1}\cdots p_{20+2\lambda}]}^{T}$ (9)

among them:

$p_{u}=\left\{ \begin{aligned} \begin{matrix} \frac{f_{u}}{\sum_{i=1}^{20} f_{i}+w\sum_{j=1}^{2\lambda} \tau_{j}}, & 1\leq u\leq20 \end{matrix} \\ \begin{matrix} \frac{w\tau_{u}}{\sum_{i=1}^{20} f_{i}+w\sum_{j=1}^{2\lambda} \tau_{j}}, & 20+1\leq u\leq20+2\lambda\end{matrix} \end{aligned} \right.$(10)

Where $f_{i}\left( i=1,2,3,\cdots,20 \right)$ is the normalized frequency of occurrence of 20 natural amino acids in protein P. The parameter λ is an integer representing the highest count of correlations along the protein sequence; w is a weighting factor ranging from 0 to 1. $\tau_{j}$ is the *j-th* sequence related factor, reflecting the sequence order correlation between all the most adjacent residues in the protein sequence, defined as:

$\left\{ \begin{aligned} \begin{matrix} \tau_{1}=\frac{1}{L-1}\sum_{i=1}^{L-1} H_{i,i+1}^{1} \\ \tau_{2}=\frac{1}{L-1}\sum_{i=1}^{L-1} H_{i,i+1}^{2} \\ \tau_{3}=\frac{1}{L-2}\sum_{i=1}^{L-2} H_{i,i+2}^{1} \end{matrix} \\ \begin{matrix} \tau_{4}=\frac{1}{L-2}\sum_{i=1}^{L-2} H_{i,i+2}^{2} \\ \cdots\end{matrix} \\ \begin{matrix} \tau_{\lambda}=\frac{1}{L-\lambda}\sum_{i=1}^{L-\lambda} H_{i,i+\lambda}^{1} \\ \tau_{\lambda}=\frac{1}{L-\lambda}\sum_{i=1}^{L-\lambda} H_{i,i+\lambda}^{2} \end{matrix} \end{aligned} \right. ,\lambda<L$(11)

Where $H_{i,j}^{1}$ and $H_{i,j}^{2}$ are correlation functions of hydrophobicity and hydrophilicity:

${H_{i,j}^{1}=h^{1}(R_{i})\cdot h^{1}(R_{j}) \atop H_{i,j}^{2}=h^{2}(R_{i})\cdot h^{2}(R_{j})}$(12)

Here $h^{1}(R_{i})$ and $h^{2}(R_{i})$ are the hydrophobic and hydrophilic values of the *i-th* ($i=1,2,3,\cdots,L)$) amino acids in the protein segment.

## References

1. Saethang T, Payne DM, Avihingsanon Y, Pisitkun T: **A machine learning strategy for predicting localization of post-translational modification sites in protein-protein interacting regions**. *BMC Bioinformatics* 2016, **17**(1):307.

2. Su MG, Huang KY, Lu CT, Kao HJ, Chang YH, Lee TY: **topPTM: a new module of dbPTM for identifying functional post-translational modifications in transmembrane proteins**. *Nucleic Acids Res* 2014, **42**(Database issue):D537-545.

3. Chen Z, Chen YZ, Wang XF, Wang C, Yan RX, Zhang Z: **Prediction of ubiquitination sites by using the composition of k-spaced amino acid pairs**. *Plos One* 2011, **6**(7):e22930.

4. Wuyun Q, Zheng W, Zhang Y, Ruan J, Hu G: **Improved Species-Specific Lysine Acetylation Site Prediction Based on a Large Variety of Features Set**. *Plos One* 2016, **11**(5):e0155370.

5. Kao HJ, Weng SL, Huang KY, Kaunang FJ, Hsu JB, Huang CH, Lee TY: **MDD-carb: a combinatorial model for the identification of protein carbonylation sites with substrate motifs**. *Bmc Syst Biol* 2017, **11**(Suppl 7):137.

6. Chang WC, Lee TY, Shien DM, Hsu JB, Horng JT, Hsu PC, Wang TY, Huang HD, Pan RL: **Incorporating support vector machine for identifying protein tyrosine sulfation sites**. *J Comput Chem* 2009, **30**(15):2526-2537.

7. Wong YH, Lee TY, Liang HK, Huang CM, Wang TY, Yang YH, Chu CH, Huang HD, Ko MT, Hwang JK: **KinasePhos 2.0: a web server for identifying protein kinase-specific phosphorylation sites based on sequences and coupling patterns**. *Nucleic Acids Res* 2007, **35**(Web Server issue):W588-594.

8. Yu CS, Chen YC, Lu CH, Hwang JK: **Prediction of protein subcellular localization**. *Proteins* 2006, **64**(3):643-651.

9. Garbuzynskiy SO, Lobanov MY, Galzitskaya OV: **FoldAmyloid: a method of prediction of amyloidogenic regions from protein sequence**. *Bioinformatics* 2010, **26**(3):326-332.

10. Suo SB, Qiu JD, Shi SP, Sun XY, Huang SY, Chen X, Liang RP: **Position-specific analysis and prediction for protein lysine acetylation based on multiple features**. *Plos One* 2012, **7**(11):e49108.

11. Li TT, Du PF, Xu NF: **Identifying Human Kinase-Specific Protein Phosphorylation Sites by Integrating Heterogeneous Information from Various Sources**. *Plos One* 2010, **5**(11).

12. Chou KC: **Prediction of protein cellular attributes using pseudo-amino acid composition**. *Proteins* 2001, **43**(3):246-255.

13. Liu B, Wu H, Chou K-C: **Pse-in-One 2.0: An Improved Package of Web Servers for Generating Various Modes of Pseudo Components of DNA, RNA, and Protein Sequences**. *Natural Science* 2017, **09**(04):67-91.
