# Supplementary material for: Prediction and analysis of multiple protein lysine modified sites based on conditional wasserstein generative adversarial networks
Source: BMC Bioinformatics. 2021 Mar 31;22:171. doi: 10.1186/s12859-021-04101-y (PMC8010967; doi:10.1186/s12859-021-04101-y)
Supplement: Supplementary file 2 — Additional file 2. S2: CWGAN generative model. [file 12859_2021_4101_MOESM2_ESM.docx]

**CWGAN**

In this paper, the tag data is considered in the input framework GAN, so the CGAN is used for tagged simulation training. According to the formula $\max_{D} V\left( G,D \right)=V\left( G,D^{*} \right) =-2log2+2JS\left( P_{data}\left( x \right)||P_{G}\left( x \right) \right)$, if the original GAN wants to get the optimal discriminator(D), it needs to achieve the loss relating to JS divergence of the real distribution $P_{data}\left( x \right)$ and the simulated distribution $P_{G}\left( x \right)$ to be the smallest. However, if there is no overlap or minimal overlap between their distributions, the JS divergence may be a fixed constant, then when the gradient is lowering, it will be 0. For the generator (G), if the gradient is 0, the gradient information cannot be obtained and the network parameters cannot be optimized. So, it is unreasonable to use JS divergence to optimize, and initializing the simulated distribution of G randomly may easily cause non-overlap with the real data.

Before introduce CWGAN, first, CGAN’s formula is as follows

$$V=E_{x\sim P_{data}}\left[ logD\left( x\mid y \right) \right]+E_{x\sim P_{G}}\left[ log\left( 1-D\left( x\mid y \right) \right) \right]$$

Next, introduce the Wasserstein distance. Wasserstein distance is also called the Earth-Mover distance (EM distance) and is used to measure the distance between two distributions:

$$W\left( p,q \right)=\inf_{\gamma\epsilon\prod\left( p,q \right)}E_{\left( x_{p},x_{q} \right)\sim\gamma}\left[ \left\| x_{p}-x_{q} \right\| \right]$$

$\prod\left( p,q \right)$ is a collection of all possible joint distributions in which $p$ and $q$ distributions are combined. For each possible joint distribution $\gamma$, a sample $x_{p}$ and $x_{q}$ can be obtained from the middle sample $\left( x_{p},x_{q} \right)\sim\gamma$, and the distance $\left\| x_{p}-x_{q} \right\|$ the sample can be calculated, so the expected value $E_{\left( x_{p},x_{q} \right)\sim\gamma}\left[ \left\| x_{p}-x_{q} \right\| \right]$ of the sample to the distance can be calculated under the joint distribution $\gamma$. The lower bound $\inf_{\gamma\epsilon\prod\left( p,q \right)}E_{\left( x_{p},x_{q} \right)\sim\gamma}\left[ \left\| x_{p}-x_{q} \right\| \right]$ that can be expected for this in all possible joint distributions is the Wasserstein distance.

Introducing CWGAN (Conditional Wasserstein Generation Adversarial Network, CGAN under Wasserstein’s method), adding noise to the simulated data and the real data to make them overlap, so that the JS divergence is effective and the gradient disappearing problem is solved, simultaneously replacing the JS divergence with the Wasserstein’s distance. The advantage of CWGAN is that it can also play a role without overlap. There is no training instability in CWGAN, basically no slipping pattern to ensure the generation of diverse simulation data, and a simple multi-layer fully connected network complex network structures can simulate raw data very well.

There are some main improvements in the network. CWGAN deletes the sigmoid function of the last layer of D. The loss function for G and D no longer uses logarithmic transformation. It uses the clip function to update the function and replace Adam with the RMSProp optimization method

To deal with the imbalance between categories, this paper uses the CGAN algorithm with Wasserstein distance.
