# Supplementary material for: Prediction and analysis of multiple protein lysine modified sites based on conditional wasserstein generative adversarial networks
Source: BMC Bioinformatics. 2021 Mar 31;22:171. doi: 10.1186/s12859-021-04101-y (PMC8010967; doi:10.1186/s12859-021-04101-y)
Supplement: Supplementary file 3 — Additional file 3. S3: Supplementary tables. [file 12859_2021_4101_MOESM3_ESM.docx]

Table 1-The Confusion matrix table in the 10-fold Cross-validation

| T/P | S_1_ | S_2_ | S_3_ | S_4_ | S_5_ | S_6_ | S_7_ |
| --- | --- | --- | --- | --- | --- | --- | --- |
| S_1_ | 2,274 | 26 | 25 | 1 | 2 | 0 | 216 |
| S_2_ | 185 | 2,074 | 18 | 20 | 69 | 9 | 138 |
| S_3_ | 228 | 76 | 1,985 | 36 | 105 | 19 | 128 |
| S_4_ | 143 | 62 | 47 | 2,139 | 66 | 31 | 70 |
| S_5_ | 201 | 17 | 20 | 3 | 2,240 | 5 | 76 |
| S_6_ | 186 | 48 | 24 | 36 | 45 | 2,092 | 76 |
| S_7_ | 56 | 0 | 0 | 0 | 1 | 2 | 2,516 |

Table 2-The DeLong test results in the 10-fold Cross-validation

| DeLong's test | S_1_ | S_2_ | S_3_ | S_4_ | S_5_ | S_6_ | S_7_ |
| --- | --- | --- | --- | --- | --- | --- | --- |
| **PCC+CWGAN+RF** vs RF | **< 2.2e-16** | **< 2.2e-16** | **< 2.2e-16** | **< 2.2e-16** | **< 2.2e-16** | **< 2.2e-16** | **< 2.2e-16** |
| **PCC+CWGAN+RF** vs PCC+RF | **0.0008** | **< 2.2e-16** | **< 2.2e-16** | **< 2.2e-16** | **< 2.2e-16** | **< 2.2e-16** | **1.374e-11** |
| **PCC+CWGAN+RF** vs PCC+CGAN+RF | **2.213e-06** | **0.0149** | 0.0528 | **0.00739** | 0.3199 | **0.0423** | 0.5634 |

Table 3-Confusion matrix of independent test.

| T/P | S_1_ | S_2_ | S_3_ | S_4_ | S_5_ | S_6_ | S_7_ |
| --- | --- | --- | --- | --- | --- | --- | --- |
| S_1_ | 575 | 7 | 8 | 1 | 0 | 1 | 49 |
| S_2_ | 60 | 546 | 9 | 3 | 21 | 2 | 31 |
| S_3_ | 42 | 13 | 478 | 5 | 35 | 6 | 29 |
| S_4_ | 38 | 14 | 16 | 519 | 18 | 8 | 14 |
| S_5_ | 59 | 4 | 3 | 1 | 535 | 0 | 21 |
| S_6_ | 57 | 14 | 16 | 11 | 12 | 555 | 13 |
| S_7_ | 6 | 0 | 0 | 0 | 0 | 0 | 604 |

Table 4- The DeLong test results in the independent test

| DeLong's test | S_1_ | S_2_ | S_3_ | S_4_ | S_5_ | S_6_ | S_7_ |
| --- | --- | --- | --- | --- | --- | --- | --- |
| **PCC+CWGAN+RF** vs RF | **5.9e-12** | **1.4e-07** | **2.48e-13** | **2.14e-11** | **6.88e-06** | **0.0003** | **1.51e-13** |
| **PCC+CWGAN+RF** vs PCC+RF | 0.06257 | **1.67e-11** | **5.37e-14** | **8.35e-10** | **3.47e-06** | **1.68e-05** | **1.16e-05** |
| **PCC+CWGAN+RF** vs PCC+CGAN+RF | **2.141e-06** | 0.0798 | **0.0114** | 0.2715 | 0.5324 | 0.2662 | 0.1862 |

Table 5- Comparisons of number of enrolled proteins and modification sites


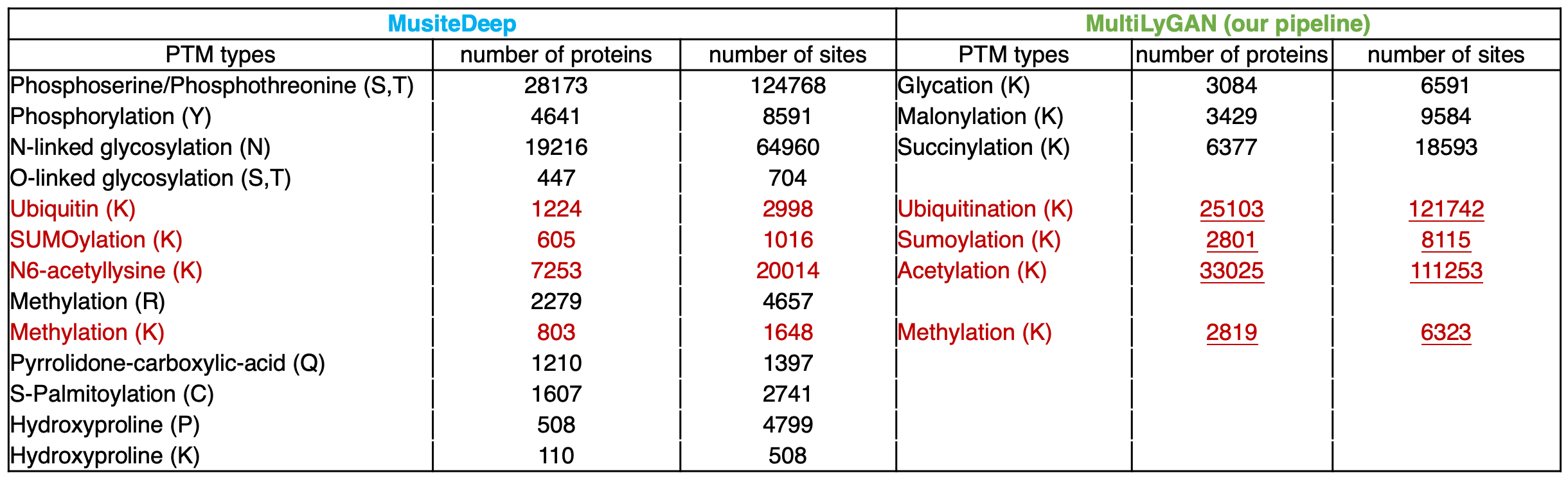


Table 6-The number of each modification type

| Dataset | Number |
| --- | --- |
| $S_{1}$(Ace) | 3114 |
| $S_{2}$(Glyca) | 1399 |
| $S_{3}$(Malon) | 1224 |
| $S_{4}$(Meth) | 1147 |
| $S_{5}$(Succ) | 1645 |
| $S_{6}$(Sumo) | 1174 |
| $S_{7}$(Ubiq) | 3185 |

Table 7-The number of each feature (*L*=17)

| Feature Construction | Dimension |
| --- | --- |
| AAindex | 238 |
| CKSAAP | 1200 |
| PWM | 17 |
| Reduced Alphabet | 136 |
| FoldAmyloid | 17 |
| BE | 340 |
| PC-PseAAC | 36 |
| SC-PseAAC | 52 |
| Structure | 323 |
| Total | 2359 |

Table 8- Euclidean distance between simulated samples and real samples with different parameters.

| Hidden dim | Learning  rate | S_1_ | S_2_ | S_3_ | S_4_ | S_5_ | S_6_ | Mean |
| --- | --- | --- | --- | --- | --- | --- | --- | --- |
| 64 | 0.01 | 0.0443 | 0.0399 | 0.0453 | 0.0403 | 0.0420 | 0.0420 | 0.0423 |
| 64 | 0.0001 | 0.0189 | 0.0118 | 0.0101 | 0.0110 | 0.0094 | 0.0129 | 0.0123 |
| 64 | 0.00001 | 0.0167 | 0.0123 | 0.0105 | 0.0113 | 0.0097 | 0.0144 | 0.0125 |
| 128 | 0.001 | 0.0349 | 0.0338 | 0.0350 | 0.0341 | 0.0339 | 0.0338 | 0.0343 |
| **128** | **0.0001** | 0.0163 | **0.0093** | **0.0089** | 0.0102 | **0.0090** | 0.0098 | **0.0106** |
| 128 | 0.00001 | 0.0161 | 0.0116 | 0.0101 | 0.0108 | 0.0102 | 0.0136 | 0.0121 |
| 256 | 0.001 | 0.0356 | 0.0349 | 0.0362 | 0.0360 | 0.0353 | 0.0362 | 0.0357 |
| 256 | 0.0001 | **0.0158** | 0.0095 | 0.0101 | **0.0101** | 0.0092 | **0.0097** | 0.0107 |
| 256 | 0.00001 | 0.0165 | 0.0124 | 0.0097 | 0.0109 | 0.0096 | 0.0144 | 0.0122 |

Table 9-The parameters of CWGAN

| Parameter | Value |
| --- | --- |
| Learning rate | 1×e^-4^ |
| Number of layers of G | 3 |
| Number of nodes per G layer | 1,498/ 128/ 1 |
| Number of layers of D | 3 |
| Number of nodes per D layer | 101/ 128/ 1,497 |
| Small batch size | 64 |
| Number of iterations | 50,000 |
| Optimization function | RMSProp |
| Clip | 0.01 |

Table 10-The parameters of random forest

| Parameter | Value |
| --- | --- |
| Subtree number | 50 |
| Subtree type | CART tree |
| Metrics | Gini index |
| Maximum characteristic number | Naught |
| Depth | No limit |


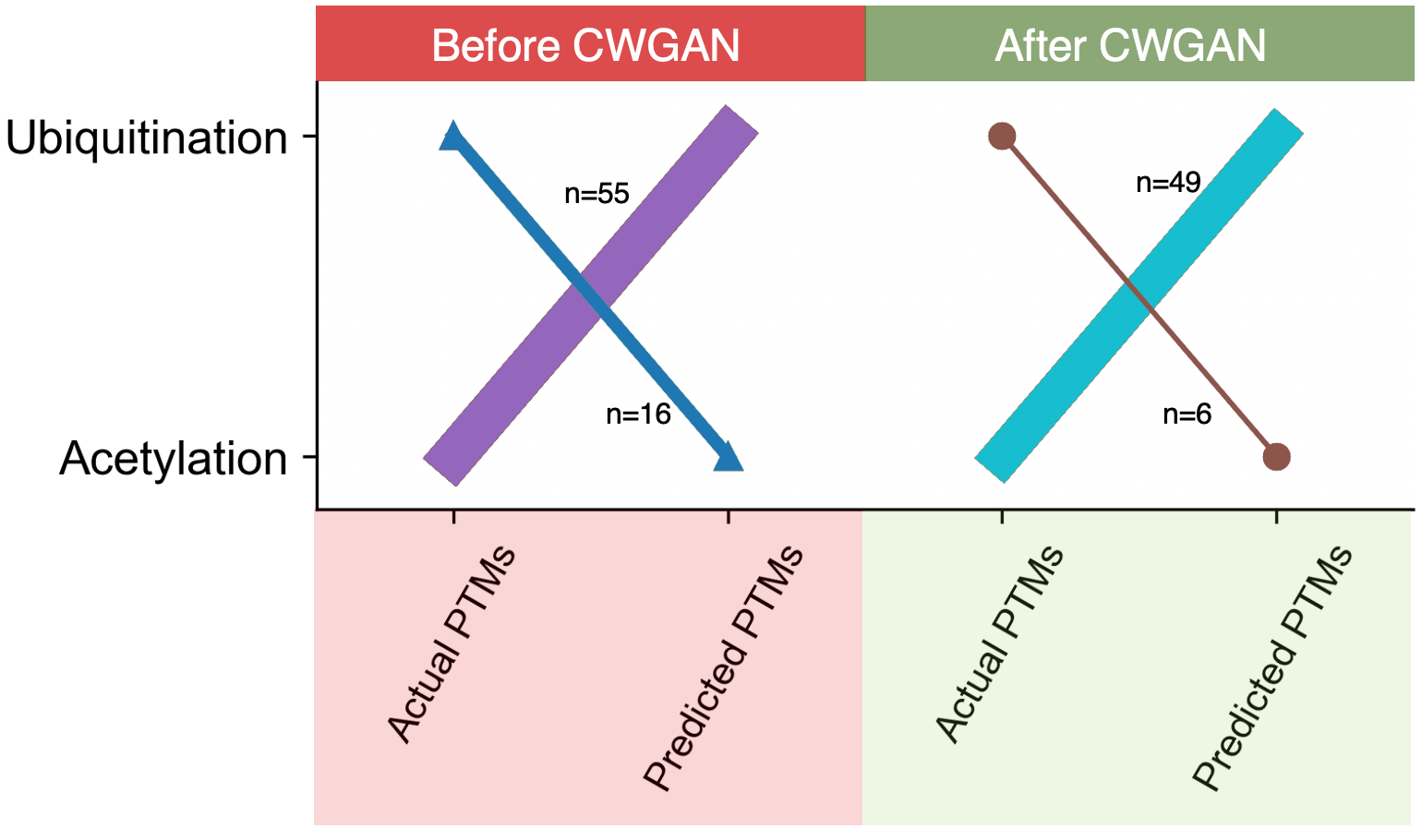


Figure 1-The comparison of two mislabeled PLMs between before CWGAN and after CWGAN.
