# Supplementary material for: Prediction and analysis of multiple protein lysine modified sites based on conditional wasserstein generative adversarial networks
Source: BMC Bioinformatics. 2021 Mar 31;22:171. doi: 10.1186/s12859-021-04101-y (PMC8010967; doi:10.1186/s12859-021-04101-y)
Supplement: Supplementary file 4 — Additional file 4. S4: Classification system indicators. [file 12859_2021_4101_MOESM4_ESM.docx]

## Measurements of performance

**Two-classification system indicator**

The common indicators of a binary classification system [1, 2] are:

$\left\{ \begin{matrix} Sn=\frac{\mathrm{TP}}{TP+FN} \\ Sp=\frac{\mathrm{TN}}{TN+FP} \\ Acc=\frac{TP+TN}{TP+TN+FP+FN} \\ MCC=\frac{\left( \mathrm{TP}\times\mathrm{TN} \right)-(FP\times FN)}{\sqrt{(TP+FP)(TP+FN)(TN+FP)(TN+FN)}} \end{matrix} \right.$ (1)

TP (true positive) is the number of correctly predicted modified fragments, TN (true negative) is the number of correctly predicted nonmodified fragments, FP (false positive) is the number of incorrectly predicted nonmodified fragments and FN (false negative) is the quantity of incorrectly predicted modified fragments. Sn, Sp, Acc and MCC are sensitivity, specificity, accuracy and Mathew correlation coefficient, respectively.

The other two important indicators, the ROC curve (receiver operating characteristic curve) and AUC (area under the ROC curve), are also used to evaluate the performance of different PTM binary classification predictors. AUC is used to reflect the probabilities of predictive performance.

**Multi-classification system indicator**

Given a multiclass problem, the sample set is S=(s_*i*:1≤*i*≤S), which is divided into N categories, the real category label of each sample is recorded as TC(s), and the prediction category label is recorded as PC(s). Acc (Accuracy), CEN (Confusion Entropy), MCC (Matthews Correlation Coefficient), Cross-validation error rate (E_C_) and independent test error rate (E_I_) are the common measurements in multiclassification. The confusion matrix formula is as follows:

$C_{ij}=\left| \left\{ s\in S:\mathrm{TC}\left( s \right)=i and PC\left( s \right)=j \right\} \right|, 1\leq i\leq N ,1\leq j\leq N$ (2)

where $C_{ij}$ is an item of the *i*-th row *j*-column in the $C (N\times N)$ matrix, indicating that the number of the *i*-th sample is predicted as the *j*-th category. The Acc and CEN are calculated as follows:

$\left\{ \begin{matrix} \mathrm{Acc}=\frac{\sum_{k=1}^{N} C_{kk}}{\sum_{i,j=1}^{N} C_{ij}} \\ CEN=-\sum_{j=1}^{N} P_{j}\sum_{\begin{aligned} k=1 \\ k\neq j \end{aligned}}^{N} ({P_{jk}^{j}log}_{2\left( N-1 \right)}\left( P_{jk}^{j} \right)+P_{kj}^{j}{log}_{2\left( N-1 \right)}\left( P_{kj}^{j} \right)) \\ MCC=\frac{cov\left( X,Y \right)}{\sqrt{cov\left( X,X \right)．cov\left( Y,Y \right)}} \\ =\frac{\sum_{k,l,m=1}^{N} C_{kk}C_{ml}-C_{lk}C_{km}}{\sqrt{\sum_{k=1}^{N} (\sum_{l=1}^{N} C_{lk})(\sum_{f,g=1,f\neq k}^{N} C_{gf})}\sqrt{\sum_{k=1}^{N} (\sum_{l=1}^{N} C_{kl})(\sum_{f,g=1,f\neq k}^{N} C_{fg})}} \end{matrix} \right.$ (3)

where $P_{j}=\frac{\sum_{k=1}^{N} (C_{jk}+C_{kj})}{2\sum_{k,l=1}^{N} (C_{kl})}$, $P_{ij}^{j}=\frac{C_{ij}}{\sum_{k=1}^{N} \left( C_{jk}+C_{kj} \right)},(i\neq j,i,j=1,2,\cdots N)$ and

$P_{ij}^{i}=\frac{C_{ij}}{\sum_{k=1}^{N} (C_{ik}+C_{ki})},i\neq j,i,j=1,2,\cdots N$. When $N>2$, the range of CEN is [0,1]. When all samples are correctly classified, CEN is 0. When all samples are classified incorrectly, CEN equals 1. Matthews Correlation Coefficient (MCC) is an important indicator of multiclassification problems. We define two matrices as X and Y, respectively. When the sample s is predicted to be *n*th $(PC\left( s \right)=n)$, $X_{sn}=1$, otherwise, $X_{sn}=0$. When the real category label of the sample is *n*th ($(TC\left( s \right)=n)$), $Y_{sn}=1$, otherwise, $Y_{sn}=0$.

$\left\{ \begin{matrix} X_{sn}={(\delta_{\mathrm{PC}\left( s \right),n})}_{sn} \\ Y_{sn}={(\delta_{\mathrm{TC}\left( s \right),n})}_{sn} \end{matrix} \right.$ (4)

where $\delta_{ij}$ is the indicative function:

$\delta_{ij}=\left\{ \begin{matrix} 1 for i=j \\ 0 for i\neq j \end{matrix} \right.$ (5)

where $C_{kk}=\left| \left\{ s\in S: X_{sk}=Y_{sk}=1 \right\} \right|=\sum_{s=1}^{S} X_{sk}Y_{sk}$. When $k\neq l$，$C_{kl}=\left| \left\{ s\in S: X_{sl}=1 and Y_{sk}=1 \right\} \right|$.

Cross-validation error rate (E_C_) and independent test error rate (E_I_) can be defined as:

$\left\{ \begin{aligned} E_{C}=\frac{\alpha_{C}}{S_{C}}\times100 \\ E_{I}=\frac{\alpha_{I}}{S_{I}}\times100 \end{aligned} \right.$ (6)

where $S_{C}$ and $S_{I}$ represent the number of samples used for training and independent test, respectively, and $\alpha_{C}$ and $\alpha_{I}$ represent the number of samples that are classified incorrectly in the cross-validation and independent test, respectively.

**References**

1. Suo SB, Qiu JD, Shi SP, Sun XY, Huang SY, Chen X, Liang RP: **Position-specific analysis and prediction for protein lysine acetylation based on multiple features**. *Plos One* 2012, **7**(11):e49108.

2. Li S, Li H, Li M, Shyr Y, Xie L, Li Y: **Improved prediction of lysine acetylation by support vector machines**. *Protein Pept Lett* 2009, **16**(8):977-983.
